# Validation of three geolocation strategies for health-facility attendees for research and public health surveillance in a rural setting in western Kenya

**DOI:** 10.1017/S0950268814000946

**Published:** 2014-05-01

**Authors:** G. H. STRESMAN, J. C. STEVENSON, C. OWAGA, E. MARUBE, C. ANYANGO, C. DRAKELEY, T. BOUSEMA, J. COX

**Affiliations:** 1 Department of Immunology & Infection, Faculty of Infectious & Tropical Diseases, London School of Hygiene & Tropical Medicine, London, UK; 2 Department of Disease Control, Faculty of Infectious & Tropical Diseases, London School of Hygiene & Tropical Medicine, London, UK; 3 Kenya Medical Research Institute, Centre for Global Health Research, Kisumu, Kenya; 4 Johns Hopkins Malaria Research Institute, Johns Hopkins Bloomberg School of Public Health, Baltimore, MD, USA; 5 Radboud University Nijmegen Medical Centre, Nijmegen, The Netherlands

**Keywords:** Infectious disease epidemiology, spatial modelling, surveillance

## Abstract

Understanding the spatial distribution of disease is critical for effective disease control. Where formal address networks do not exist, tracking spatial patterns of clinical disease is difficult. Geolocation strategies were tested at rural health facilities in western Kenya. Methods included geocoding residence by head of compound, participatory mapping and recording the self-reported nearest landmark. Geocoding was able to locate 72·9% [95% confidence interval (CI) 67·7–77·6] of individuals to within 250 m of the true compound location. The participatory mapping exercise was able to correctly locate 82·0% of compounds (95% CI 78·9–84·8) to a 2 × 2·5 km area with a 500 m buffer. The self-reported nearest landmark was able to locate 78·1% (95% CI 73·8–82·1) of compounds to the correct catchment area. These strategies tested provide options for quickly obtaining spatial information on individuals presenting at health facilities.

## INTRODUCTION

Many infectious diseases show microepidemiological geographical variation. Outbreaks of (emerging) infectious diseases may be geographically confined or start in small pockets that later give rise to larger outbreaks [1–4]. For endemic infectious diseases with stable disease transmission, considerable geographical heterogeneity in the intensity of transmission has been described [2, 5–8]. Geographical variation for both epidemic and endemic infectious disease occurrence has important public health consequences. Identifying regions with higher disease burden can facilitate cost-effective prioritization of control efforts [9–11]. Within regions, identifying areas of persistent and intense transmission may prevent outbreaks of disease that spread from these areas and support disease elimination strategies when overall disease occurrence has declined [2, 12, 13]. To allow spatial targeting of disease control efforts, attributing a geographical location to each disease occurrence is ideal, and the minimum number required for accurate monitoring is likely to be disease specific [9, 14, 15].

Given adequate address information, automated geocoding software packages can generate accurate spatial coordinate data for a large proportion of individuals [16, 17], thereby providing a basis for the spatial analysis of disease transmission [18–20]. In circumstances where formal address data are unavailable or privacy concerns limit the use of precise spatial locations, other approaches have been used to obtain geographical information on incident cases. Catchment areas of, for example, community pharmacies or general practitioners have been used for describing spatial patterns in disease occurrence [6, 15, 20–22]. In areas with well-developed public health infrastructure, catchment areas tend to be well defined and sufficiently small to allow a meaningful attribution of localities to clinical cases based on the facility they attended [20, 22]. Geolocation approaches are likely to have less utility for resource-poor settings where formal address systems are commonly unavailable and where health-facility catchment areas are relatively large and poorly defined [5, 23, 24]. Alternative approaches to geolocation strategies are needed in such settings.

Two of the most commonly used geolocation strategies for rural resource-poor environments are distributing compound ID cards after an enumeration exercise or actively visiting compounds and geolocating the area of residence for individuals of interest [25]. Although these methods provide accurate spatial information, they are not operationally attractive outside research settings [10, 21, 25]. Approaches that can be implemented without the need for house-to-house visits would facilitate the incorporation of spatial information into routine data collection and public health planning at the local level. If this can be done with sufficient precision it would support the identification of local-level disease heterogeneity [5, 18, 25].

Here, we examine the accuracy and precision of three approaches to geolocate health facility attendees in a rural area of western Kenya: geocoding on name of head of compound, participatory mapping using satellite imagery, and attributing participants to the catchment area of the self-reported nearest landmark.

## METHODS

### Study site

**Fig. 1:**
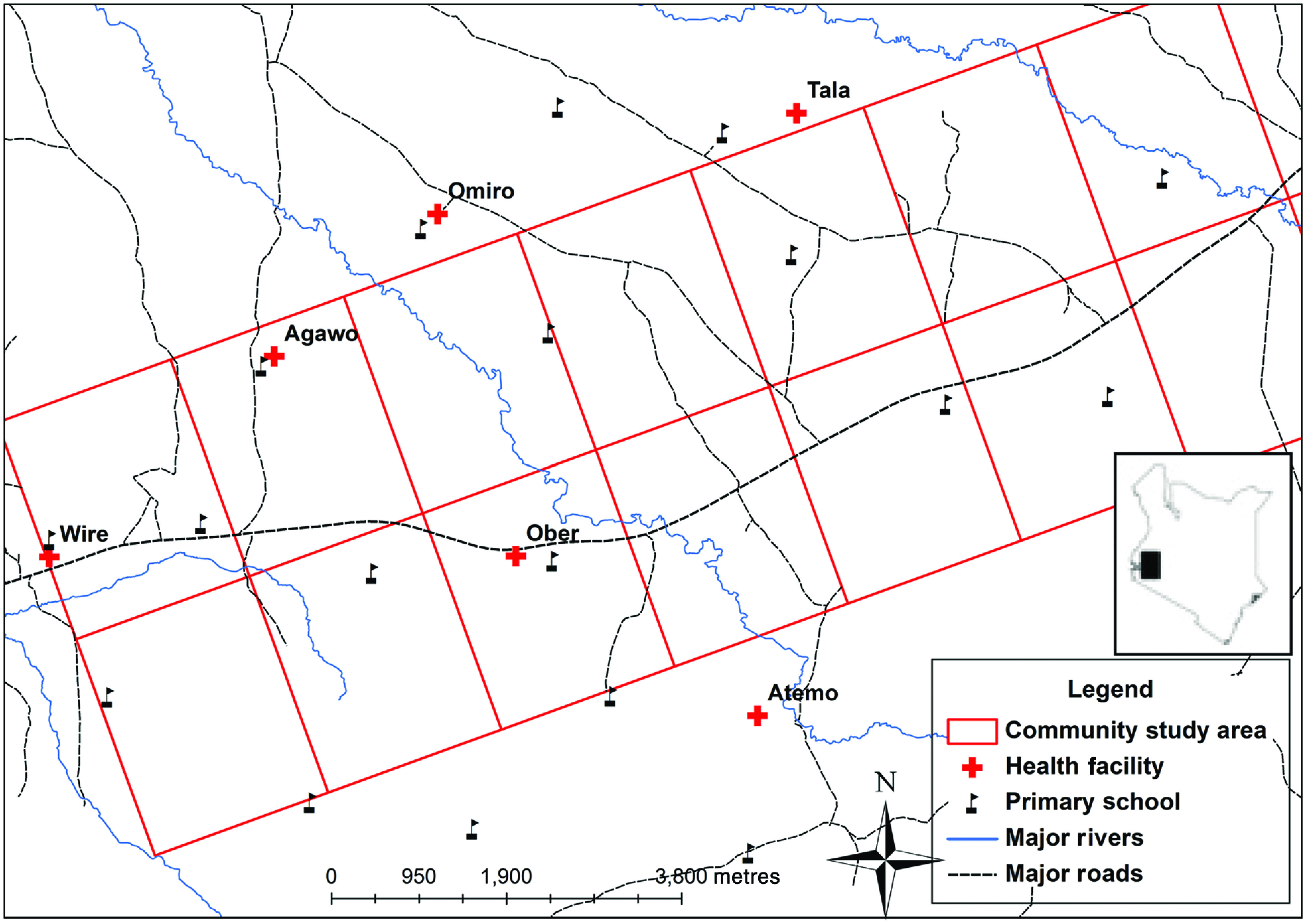
[*colour online*]. Map of the study area, Rachuonyo South, Kenya (2011–2012), showing the main roads (dashed lines), rivers (solid lines), location of schools (flags) and health facilities (crosses).

The study was conducted in a rural area of Rachuonyo South district, Nyanza Province in the western Kenyan highlands that spans about 300 km^2^. There is one main road that runs through the area and the landscape consists of rolling hills and several large rivers (Fig. 1). The population mostly comprises people from the Luo ethnic group whose main occupation is subsistence agriculture. Compounds typically comprise extended families living in proximity to their fields or in multi-unit structures in the few, more urban, market centres [26].

Five rural health facilities were identified whose catchments overlapped with community-based cross-sectional surveys being performed (Fig. 1) [27]. Cross-sectional malaria surveys in the health facilities were conducted in October 2011 and in July 2012 to coincide with the bimodal seasonal peaks in malaria transmission. Four of the five health facilities were sampled during both surveys. One facility was replaced for the second year to maximize overlap with the ongoing community work. All patients and accompanying individuals attending the outpatient clinic were recruited for the survey. A questionnaire was administered to all consenting participants to obtain information on malaria indicators and their area of residence, as described below. Tracing individual compounds from health-facility attendees is a laborious and costly exercise because of the large catchment areas and inaccessible terrain and could therefore not be completed for all attendees. For operational reasons, following the facility survey, 30% of participants were randomly selected and traced to their compounds, to validate the geolocation strategies being tested. Compounds were mapped using a GPS receiver.

### Geolocation strategies

#### Method 1: Geocoding

A system of geocoding was developed to match ‘postal addresses’ to an existing spatial database. In this setting in rural Kenya, compounds are known by the name of the compound head, usually the patriarch of the family. Individuals have three names, two given and one family name. Names of the compound head were collected as part of the questionnaire at the facility. Names were matched to an existing database of names of compound heads with associated spatial coordinates collected as part of a large cross-sectional survey in the area. This community survey sampled about one third of the population [27]. As not all compounds were sampled during the community survey, the names of the three nearest neighbours were also collected at the facility to increase the probability of finding a match. This method would be useful in areas that have existing and updated registries with accompanying spatial information and could easily be applied to all scales, depending on the availability of baseline data.

Analysis was restricted to those compounds located in the area of the community survey. Names from the two databases were matched using Levenshtein's distance algorithm [28] for string matching using Stata v. 12.1 (StataCorp, USA). Possible matches, where the matching probability was ⩾80%, were checked manually. Matches were discarded if: (*a*) there was more than one compound head with the same name in either database; (*b*) if only one of the three names was recorded; or (*c*) if all three names were provided but at least one of the names did not match. This process was repeated for the names of the nearest neighbours. All likely matches were plotted in ArcGIS v. 10.1 (ESRI, USA) and the distance between the actual geolocated compound and the matched compound from the community survey was calculated. Compounds from the health-facility survey were considered successfully located if they were <250 m from the corresponding compound in the community survey. This resolution was a pragmatic choice as it was deemed an acceptable balance between accuracy and spatial resolution, as this area would only likely comprise 2 or 3 compounds.

#### Method 2: Participatory mapping

**Fig. 2.**
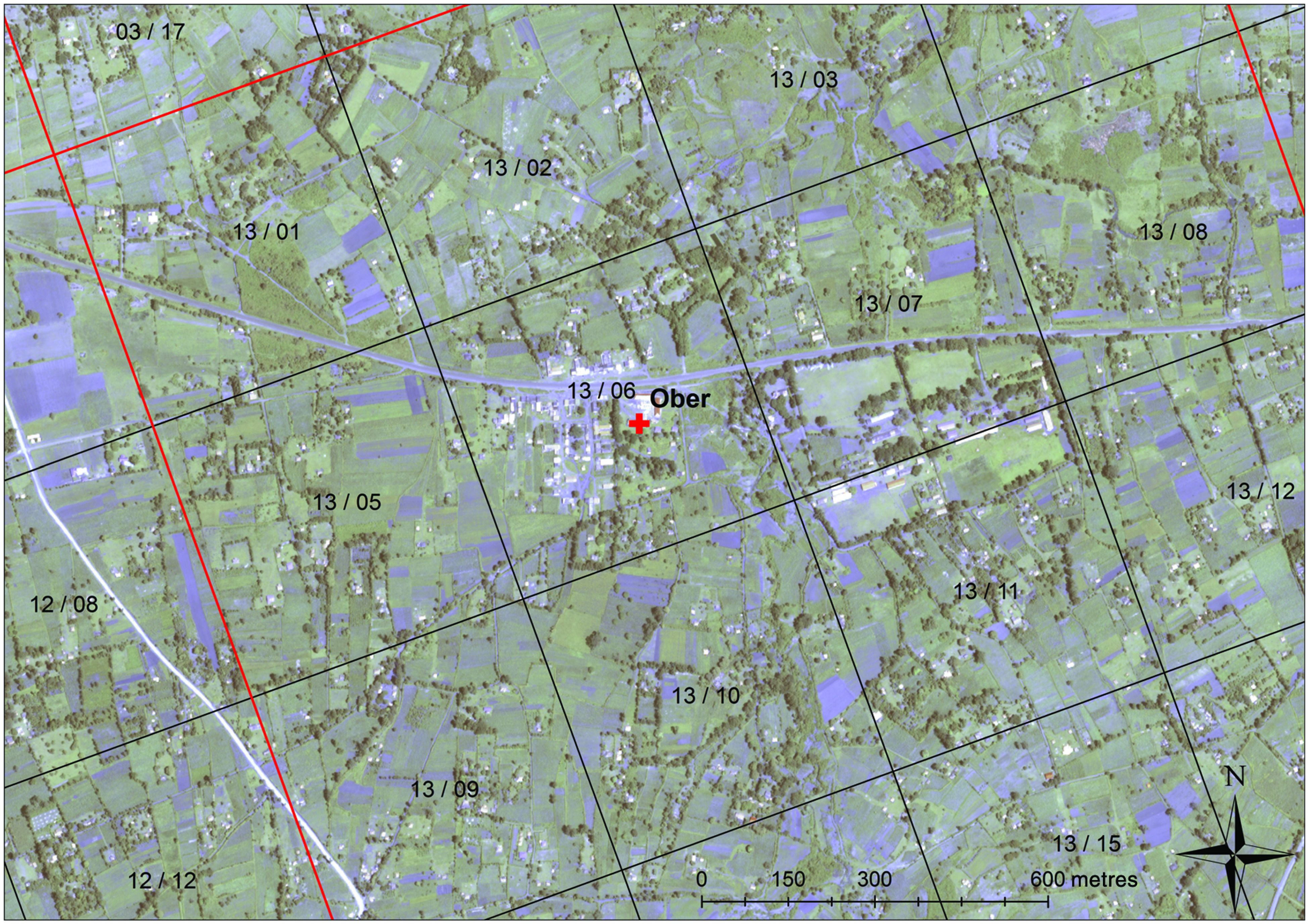
Participatory mapping example showing the grid of blocks and cells that were overlain on satellite imagery. The red lines outline the block and block numbers are shown. The cells are outlined by the black lines within each block and are counted from 1 to 20 starting with the upper left corner and counting from left to right (i.e. 13/01 to 13/20).

The second method assessed was participatory mapping, and was similar to the recently published ‘map-book’ exercise [25] and involved producing poster-sized, high-resolution satellite images (Quickbird; Digital Globe, USA) of each facility catchment area (Fig. 2). Locations of health facilities, schools, markets and other key landmarks were labelled on the image and a reference grid consisting of 2 × 2·5 km ‘blocks’ was superimposed on the area [27]. Each block comprised 20 ‘cells’, each measuring 500 × 500 m. Each block/cell combination was given a unique numeric identifier. The system (including size of polygon) was selected because it was familiar to the field workers and would provide them a better frame of reference for facilitating the exercise. As part of the participant questionnaire, the interviewer would explain the main features of the satellite map and with the participant, would attempt to locate the residence on the map and record the corresponding cell identifier. Due to the spatial resolution required to locate compounds, this approach is most applicable to local scale but could be scaled up if satellite imagery was indexed into a book-format instead of a poster.

Locations of participants followed to their compounds were plotted in ArcGIS and were classified as correctly located based on the participatory mapping exercise if they fell within the reported cell. To account for the likely edge effect with compounds located just outside a grid cell being considered incorrect, the proportion of compounds correctly identified within 500 m (one cell) or 1000 m (two cells) surrounding the reported block/cell was also calculated. The distance between the edge of the cell/buffer and the incorrectly located compounds was calculated in ArcGIS to determine the mean error associated with the approach.

#### Method 3: Nearest self-reported landmarks

The final method tested was to see if participants resided in the catchment of self-reported nearest landmarks. This approach is the most flexible and could be easily applied at all scales, given a database of the relevant landmark with accompanying spatial information is available. At the health facility, each participant was asked to name the nearest health facility, primary school, market and church to their compound. Combinations of responses were also assessed using overlapping catchment areas to increase the precision of the approach. Locations of compounds were plotted using ArcGIS and a compound was considered to be correctly located if it fell within the catchment area or intersecting catchment areas that matched the response provided at the facility.

**Fig. 3.**
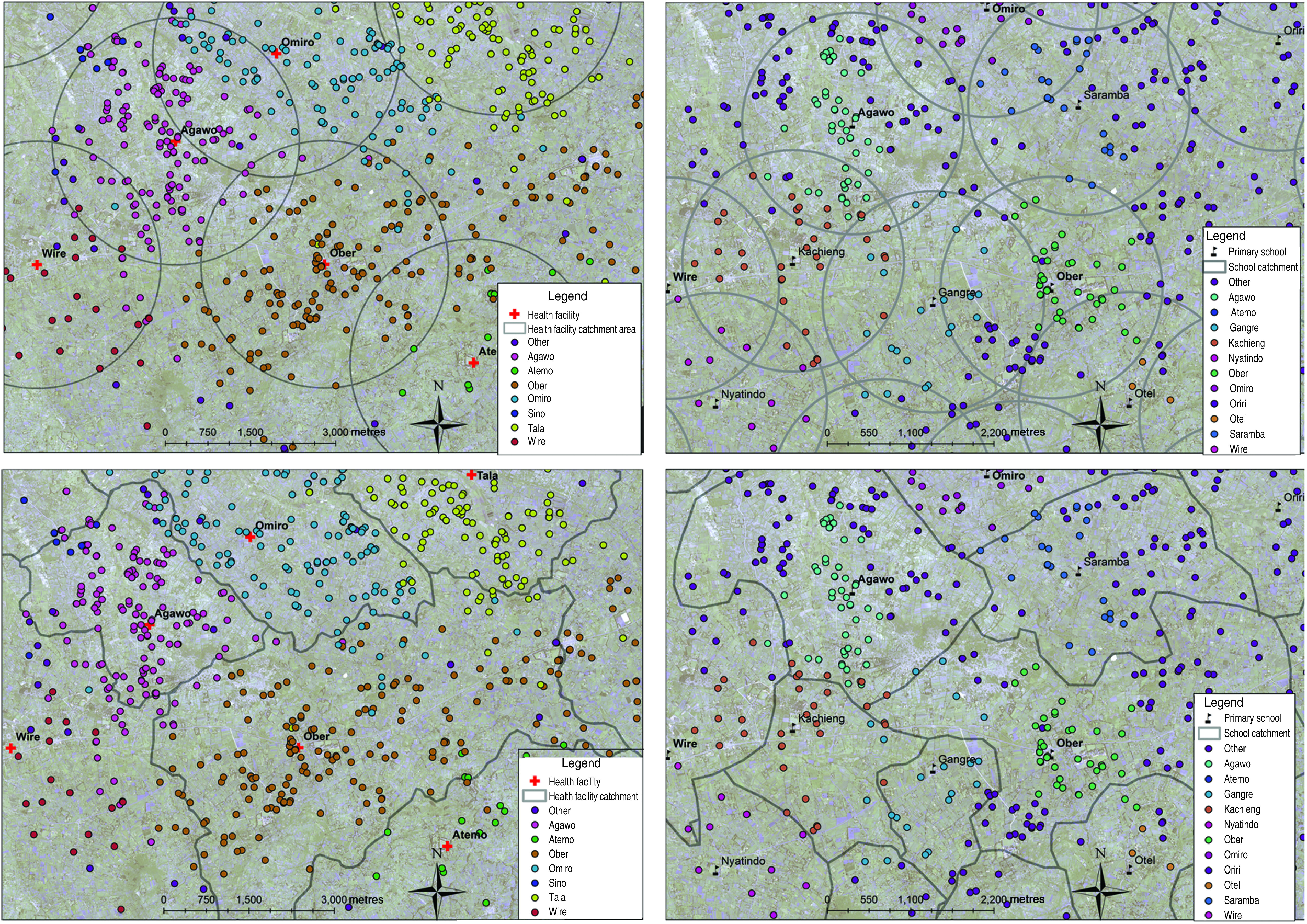
Examples of the catchment areas and the spatial distribution of responses for self reported nearest landmark for the Euclidian and cost-distance models, South Rachuonyo, Kenya, 2011–2012. (*a*) Health-facility catchment based on Euclidian distance model; (*b*) primary school catchment based on Euclidian distance model; (*c*) health-facility catchment area based on cost-distance model; (*d*) school catchment area based on cost-distance model.

Catchment areas for each type of landmark were estimated based on both Euclidian distance (straight-line) and cost distance [29, 30]. There were some missing coordinates for certain reported schools. Therefore, analysis was restricted to participants who reported residing near the schools with known coordinates. Euclidian distances were calculated using the ArcGIS Euclidian distance tool in the spatial analyst package to delineate catchment areas for both health facilities (Fig. 3*a*) and primary schools (Fig. 3*b*).

A cost-distance function to account for factors that may either impede or facilitate travel was also used to delineate landmark catchment areas. Given the gently undulating topography of the study area, it was assumed that ease and speed of travel between compounds and relevant landmarks is determined either by the presence of roads (facilitating travel) or by the presence of rivers (impeding travel). Roads and rivers in the study area were digitized using high-resolution Quickbird satellite multispectral imagery at 2·8 m resolution sharpened with a 60 cm resolution panchromatic image. Roads were classified into four categories: (1) tarred roads where the likely maximum speed is 80 km/h; (2) roads that are not tarred but where vehicles travel at a likely maximum speed of 40 km/h; (3) roads that are not tarred but accessible to a vehicle or motorbike with likely maximum speeds of 20 km/h; (4) paths not likely traversed by a vehicle but by motorbike with likely maximum speeds of 10 km/h. For all other surfaces, including walking paths or fields, a maximum speed of 5 km/h was assumed [23]. Rivers were classified as barriers to movement except where they were intersected by a road or path. The cost-distance models for both health facilities (Fig. 3*c*) and primary schools (Fig. 3*d*) were created using IDRISI software (Clark Laboratories, USA) and imported into ArcGIS for analysis.

The mean error for both methods was calculated as the distance between the border of the catchment and the location of the incorrectly located compound. The distance between each compound and the centroid of each polygon could have been calculated. However, due to the irregular shape of many of the polygons, the distance to the centroid is not be an accurate reflection of the error rate in this approach as points that are far away from the centroid but located to the correct catchment area would generate a large error rate and be misleading.

### Ethical considerations

This study was approved by the ethics committees of the London School of Hygiene and Tropical Medicine (LSHTM 5956) and the Kenya Medical Research Institute (SSC 1589). Individual informed consent was sought from all participants of the health-facility survey by signature or thumbprint accompanied with the signature of an independent witness. As defined in the Kenya national guidelines, participants aged <18 years who were pregnant, married, or a parent were considered ‘mature minors’ and consented for themselves [31].

### Data analysis

The proportion of study participants whose compounds were correctly located using each geolocation strategy of all participants that provided responses for each method and corresponding binomial 95% confidence interval (CI) was calculated. Mean error of each method was determined by calculating the distance between the actual location of the compound and edge of the identified area. Plotting the proportions for each approach against the mean area identified the optimum strategy: strategies located in the top left corner of the plot signified high precision and accuracy.

## RESULTS

Across both surveys, 3034 people were enrolled of which 830 (27%) were able to be traced back to their compounds and included in the analysis. Those that could not be traced were mainly due to running out of time and inaccurate information provided at the facility. The participants that could not be traced were evenly distributed between years and facilities.

### Method 1: Geocoding

Of the geolocated participants, 519 lived within the area of the community cross-sectional malaria survey and could be used for geolocation. Of the 328 matched compounds, 56% were successfully located using the head of compound. Of the participants that were matched, 72·9% were correctly located to within 250 m (95% CI 67·7–77·6, median distance 36·2 m). Possible reasons for why more people were not correctly matched may include people not being familiar with the full names of their neighbours or reporting different heads of compound for the same compound (e.g. the grandfather *vs*. the father of the family). The median distance from the true location to the matched compound of those that were incorrectly matched was 4440·9 m [interquartile range (IQR) 1610·1–8591·4 m].

### Method 2: Participatory mapping

Using the participatory mapping approach, 64·9% (95% CI 61·2–68·4) of 695 participants who attempted the mapping exercise were successfully located to the appropriate 2 × 2·5 km block (Table 1). When a 500 m buffer in all directions around the block was included, the proportion correctly located improved to 82% (95% CI 78·9–84·8) at the block level and from 12·4% (95% CI 10·0–15·0) to 57·1% (95% CI 53·3–60·8) at the cell level.

**Table 1.** Results of participatory mapping exercise, Rachuonyo South, Kenya, 2011–2012

	Block/cell only			>500 m buffer			>1000 m buffer		
	Mean area (km^2^)	% Correct	95% CI	Mean area (km^2^)	% Correct	95% CI	Mean area (km^2^)	% Correct	95% CI
Block	5	64·9	61·2–68·4	7·5	82·0	78·9–84·8	10·5	90·6	88·2–92·7
Cell	0·25	12·4	10·0–15·0	1	57·1	53·3–60·8	2·25	77·1	73·8–80·2

CI, Confidence interval.

However, 135 (16·3%) participants did not participate in the mapping exercise. Reasons for refusal were not recorded, but there were no differences in sex or age distributions in the populations who did and did not participate in the exercise. Of those willing to locate their residence, 61·5% were female compared to 58·9% in the unwilling group (*P* = 0·6). Similarly, the mean age in the adult populations in those unwilling to locate their residence was slightly higher at 37·9 years compared to 35·3 years in those that did attempt the exercise, although the difference was not significant (*P* = 0·3).

For compounds that were incorrectly located, the median distance to the correct block was 489 m (IQR 229–1036 m), 1036 m (IQR 737–1737), and 1737 m (IQR 1179–2728) for the block only, >500 m buffer, and >1000 m buffer, respectively. The median distance of compounds incorrectly located from the identified cells was 539 m (IQR 236–1095 m), 1055 m (IQR 737–1644) including a 500 m buffer, and 1588 m (IQR 1200–2180 m) including a 1000 m buffer. Moreover, the proportion of people that were correctly identified to a specific block or cell significantly varied per facility (block only, *P* = 0·007; >500 m, *P* = 0·003; >1000 m, *P* < 0·0001).

### Method 3: Nearest self-reported landmarks

Analysis of self-reported nearest landmarks indicated that responses for nearest market tended to predominantly consider relatively large markets, rather than smaller, local markets. In addition there was too much variability in responses concerning the nearest church, the majority of which were small establishments whose spatial coordinates had not been recorded, to conduct meaningful analysis. For these reasons only data relating to the nearest health facility and primary school were retained.

Overall, the nearest health facility and primary school were reported correctly 84·9% (95% CI 82·2–87·2) and 73·4% (95% CI 68·8–77·7) of the time, respectively, based on straight-line distance (median distance 1486 m, IQR 1008–2241 m). The use of the self-reported nearest primary school was able to locate 82·0% (95% CI 78·1–85·8) of participants' compounds to the correct Euclidian distance catchment area (mean area of 6·7 km^2^) (Table 2) with a median distance to the self-reported nearest school of 878 m (IQR 522–1234 m). The self-reported nearest health facility was able to locate 78·1% (95% CI 73·8–82·1) of compounds to an area of 12·3 km^2^. When the combination of responses was tested, the mean area reduced to 1·7 km^2^ and 48·7% (95% CI 43·6–53·6) of participants' compounds were correctly located.

**Table 2.** Results of self-reported nearest landmarks as a geolocation strategy, Rachuonyo South, Kenya, 2011–2012

	Euclidian distance			Cost distance		
	Mean area (km^2^)	% Correct	95% CI	Mean area (km^2^)	% Correct	95% CI
Health facility	14·9	73·9	70·7–76·8	36·3	77·1	74·1–80·0
Primary school	6·7	82·0	78·1–85·8	12·3	78·1	73·8–82·1
Health facility & school	1·7	48·7	43·6–53·6	3·7	72·4	67·8–76·8

CI, Confidence interval.

Next, 77·1% (95% CI 74·1–80·0) and 78·1% (95% CI 73·8–82·1) of participants were located to the correct health facility and school catchments, respectively, using the cost-distance catchment area. The combined responses were able to locate individuals based on the combination of responses with 72·4% (95% CI 67·8–76·8) of compounds successfully located to a mean area of 3·7 km^2^ (table 2).

Of those individuals who did not reside in the catchment area of the reported nearest landmark, the mean distance away from the edge of the catchment area was 1252 m (IQR 261–1899 m) for catchments based on Euclidian distance and 496 m (IQR 174–605 m) using the cost-distance model.

### Optimal geolocation approach

**Fig. 4.**
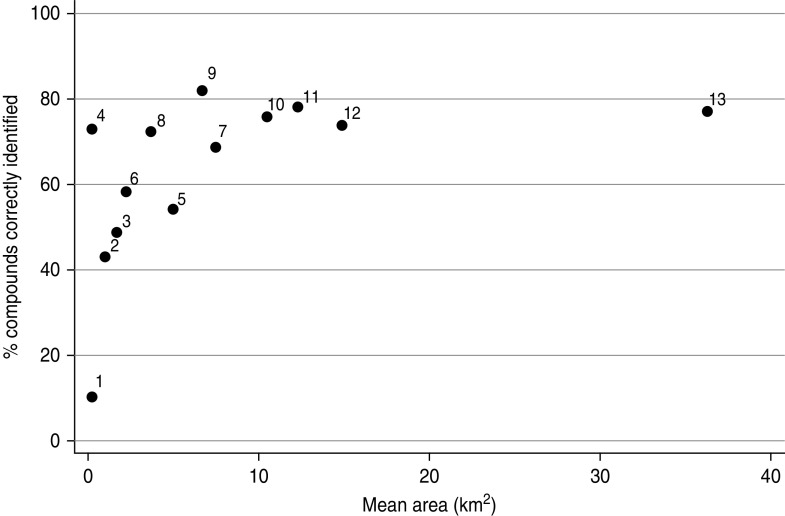
Scatter plot showing the summarized results of all geolocation strategies tested with the precision (mean area) of the approach plotted against the accuracy (% of compounds correctly located): 1, cell [participatory mapping (PM)]; 2, cell (>500 m) (PM); 3, combined health facility (HF) & primary school (PS) (Euclidian distance; ED) [nearest landmark (NL)]; 4, geocoding; 5, block (PM); 6, cell (>1000 m) (PM); 7, block (>500 m) (PM); 8, combined HF & PS (cost-distance; CD) (NL); 9, PS (ED) (NL); 10, block (>1000 m) (PM); 11, PS (CD) (NL); 12, HF (ED) (NL); 13, HF (CD) (NL).

Although not directly comparable due to the different scales, the results across all strategies showed a logarithmic relationship between mean catchment area and proportion of compounds correctly identified (Fig. 4). Points that are located in the top left corner represent the optimal combination of low mean area (high precision) and a high proportion of people correctly located using that strategy (high accuracy). The results of this analysis suggest that using the location of the nearest primary school as well as the participatory mapping with buffer was the most promising method to geolocate rural health-facility attendees in this rural study setting.

## DISCUSSION

A simple and operationally feasible way to identify the spatial occurrence of disease in rural areas where homes have no formalized address would be an extremely useful tool and could easily be employed as an operationally attractive approach to spatial disease surveillance in a wide range of settings around the world. A recent study has been conducted in Blantyre, Malawi in an urban setting [25]; however, our study is, to our knowledge, the first attempt to examine different methods to geolocate health-facility attendees in a rural area and to gauge their precision. Although strategies are not directly comparable due to the different spatial scales, the current study showed that there are options available to obtain spatial information in areas where no formal postal network exists. Results have shown that it was possible to correctly locate close to 80% of participant compounds using either a participatory mapping exercise (to 2 × 2·5 km blocks with buffer) or by using information about the nearest primary school. This is similar to the level of detection of most geocoding strategies when applied in developed countries, although the spatial resolution is not as good [17, 32]. In this study, methods based on name-matching or participatory mapping to the 500 × 500 m cell level proved to be less accurate, but are capable of greater spatial precision.

The ideal geolocation approach in a rural setting will ultimately depend on the information available, the objectives, whether it be monitoring for epidemics or planning for disease control interventions, and the required spatial precision/accuracy. The geocoding approach requires that an accurate and up-to-date list of names of compound heads is available, which is unlikely to be the case outside areas of active community-based research. The geocoding approach also relies on names recorded being complete and recorded consistently; a difficult task in busy facilities. There may also be challenges in obtaining correct information from people who may want to remain anonymous. Moreover, a systematic bias is inevitable as compounds whose head has a common name or is the head of multiple compounds will never be matched unless other variables are also considered. However, in areas where a complete database is available, through land registries for example, or if overall accuracy is less important, geocoding could provide a useful geolocation approach.

The participatory mapping exercise also has notable limitations. It requires that a map of the study area be available and that there are personnel familiar with the area capable of interpreting satellite imagery. Key features must be identifiable on the map to help orient readers. Although the age difference here was not significant, younger generations may also be more map literate than older generations. High-resolution satellite imagery can be expensive to acquire, up to several thousand US dollars [25]; however, free imagery with good resolution is becoming more widely available for even remote areas in rural and low-income settings and a similar exercise could be conducted using web-based platforms as is increasingly being utilized for disaster response [33–35]. Further, depending on the size of the area of interest, it may be possible to create a schematic map of the area using local knowledge [10].

To facilitate participatory mapping, a grid was superimposed onto the study area, leading to an edge effect whereby if a person was located just outside of the block/cell they would be classified incorrectly even though the error margin could be only a few metres. Edge effect will always be an important limitation that must be accounted for in any application of this methodology particularly when the focus is on locating residences at a precise spatial resolution. However, despite this limitation, this research has provided important insight into how the edge effect can be minimized and sensitivity increased by the addition of buffer zones. Other approaches could have been used including a hexagonal grid or larger clusters as was used in the study in Blantyre's urban slum area [25]. These approaches will likely reduce, but not completely eliminate the edge effect. Moreover, in this study, there was a significant difference in the proportion of people correctly located at each health facility and not every participant was willing to complete the exercise. This suggests that the familiarity of the interviewers with the area, their ability to read and explain the maps to local populations, and the time they have or choose to dedicate may be important determinants for success.

The use of the nearest landmark approach requires that the location of the feature in question (e.g. church, school) be known. This could be done by visiting and mapping each site using a GPS receiver, or sites could be located on a map by someone familiar with the area. National databases of the locations of such landmarks are becoming more common and therefore this limitation may be less relevant; however, to be useful, databases must be up to date and include all government, faith-based, and private facilities. In this study, people only correctly located the nearest landmark around 80% of the time and the accuracy of this approach was dependent on the definition of catchment area used. The reporting bias may be due to factors such as spatial perceptions of ‘closeness’, the density of that type of landmark in the area, or reporting known or highly frequented landmarks rather than those that are closer. Other possible landmarks that could be used include nearest chief or assistant chief, nearest shop, or nearest local transport point. In terms of defining catchment areas, both methods produced similar results [36]. The analysis using the cost-distance catchment areas showed a lower error rate based on the distance from the edge of the catchment area suggesting that this approach may be more robust. However, the utility of this approach is limited to areas with digitized travel networks, access to the required software, and the expertise to create the cost-distance surface is required.

The goals of the geolocation exercise will influence the optimum strategy. First, the ideal scale will depend on the spatial pattern of the disease and the size of the area of interest [5]. For example, if the objective was to identify foci of infections of a highly heterogeneous disease such as malaria in a low endemic or epidemic setting [7, 9, 20] then achieving higher precision would be essential. Conversely, if the distribution of sexually transmitted infections was being studied, less precision may be acceptable or even necessary to guarantee anonymity [20]. Second, the ideal strategy will depend on the purpose of geolocating cases. If it is for programmatic use such as passive public health surveillance, or to establish disease distribution at a regional or national level, then using the nearest health facility, with a larger mean catchment area may be sufficient. However, if greater precision and accuracy were required, for identification of foci for disease elimination or identifying where to implement control, for example, then knowing the exact boundaries of the catchment area or having a comprehensive postal network that can be geocoded to a high precision would be essential.

There were some limitations to this study. First, it was only feasible to trace 27% of participants to their compounds. Although this provided a large sample, it is possible that if we could have traced all individuals, the results and the conclusions on the applicability of the techniques tested may have been different. However, as the sample was a random selection, the impact on the results is expected to be minimal. Similarly, spatial coordinates were only available for the government-run primary schools in the area, thereby restricting the sample to those residing near these schools. The limited number of school locations that were available as well as the lack of covariates such as size or perception of academic rigour to include as part of delineation of catchment areas likely influenced the size of catchment areas as calculated by both approaches. However, although altered catchment area boundaries would impact both the precision and accuracy of the results, this is not likely to have a significant impact of the results.

Spatial monitoring of health-facility data has strengthened public health programmes in developed countries and facilitates conducting research with passively collected data [6, 37]. However, the ability to efficiently geolocate individuals residing in areas where no formal address network exists or where the settlement pattern is not conducive to matching individuals to specific localities is currently lacking, particularly in areas around the world where infectious disease transmission persists [5, 38]. The geolocation strategies tested as part of this research exemplify alternative options for obtaining spatial information from health-facility patients in a setting that is typical for much of rural sub-Saharan Africa and other parts of the world. Easily collected spatial information can supplement both passive and active disease surveillance to detect foci of transmission, enables the detection of outbreaks in a timely manner, and facilitates tracking of how disease spreads through the population over time [37, 39, 40]. If validated in other parts of the world, these results indicate that recording the nearest primary school or implementation of a participatory mapping exercise at rural health facilities offer potential strategies to facilitate spatial analysis of disease dynamics. Further research is needed to demonstrate their utility in a range of settings and their operational viability before formal testing in a broader operational context.
